# Berberine ameliorates DSS-induced intestinal mucosal barrier dysfunction through microbiota-dependence and Wnt/β-catenin pathway

**DOI:** 10.7150/ijbs.65476

**Published:** 2022-01-16

**Authors:** Yalan Dong, Heng Fan, Zhe Zhang, Feng Jiang, Mingyue Li, Haifeng Zhou, Weina Guo, Zili Zhang, Zhenyu Kang, Yang Gui, Zhexing Shou, Junyi Li, Rui Zhu, Yu Fu, Alexey Sarapultsev, Huafang Wang, Shanshan Luo, Ge Zhang, Desheng Hu

**Affiliations:** 1 Department of Integrated Traditional Chinese and Western Medicine, Union Hospital, Tongji Medical College, Huazhong University of Science and Technology, Wuhan 430022, China; 2 Department of Physical Examination, The Central Hospital of Wuhan, Wuhan, China; 3 Tianjin University of Traditional Chinese Medicine, Tianjin, China; 4 Department of Gastroenterology, Union Hospital, Tongji Medical College, Huazhong University of Science and Technology, Wuhan, 430022, China; 5 School of Medical Biology, South Ural State University, 454080 Chelyabinsk, Russia; 6 Institute of Hematology, Union Hospital, Tongji Medical College, Huazhong University of Science and Technology, Wuhan 430022, China; 7 Institute of Integrated Bioinfomedicine and Translational Science, School of Chinese Medicine, Hong Kong Baptist University, Hong Kong SAR 999077, China

**Keywords:** Berberine, Ulcerative colitis, Microbiota, Intestinal mucosal barrier, Wnt/β-catenin pathway

## Abstract

Ulcerative colitis (UC) is an idiopathic, chronic inflammatory disorder of the colon, and it has become one of the world-recognized medical problems as it is recurrent and refractory. Berberine (BBR) is an effective drug for UC treatment. However, the underlying mechanism and targets remain obscure. In this study, we systematically investigated the therapeutic effect and its mechanism of BBR in ameliorating DSS-induced mouse colitis. Expectedly, the colon inflammation was significantly relieved by BBR, and microbiota depletion by antibiotic cocktail significantly reversed the therapeutic effect. Further studies showed that BBR can regulate the abundance and component of bacteria, reestablish the broken chemical and epithelial barriers. Meanwhile, BBR administration dramatically decreased ILC1 and Th17 cells, and increased Tregs as well as ILC3 in colonic tissue of DSS-induced mice, and it was able to regulate the expression of various immune factors at the mRNA level. Moreover, a proteomic study revealed that Wnt/β-catenin pathway was remarkably enhanced in colonic tissue of BBR-treated mice, and the therapeutic effect of BBR was disappeared after the intervention of Wnt pathway inhibitor FH535. These results substantially revealed that BBR restores DSS-induced colon inflammation in a microbiota-dependent manner, and BBR performs its protective roles in colon by maintaining the structure and function of the intestinal mucosal barrier, regulating the intestinal mucosal immune homeostasis and it works through the Wnt/β-catenin pathway. Importantly, these findings also provided the proof that BBR serves as a potential gut microbiota modulator and mucosal barrier protector for UC prevention and therapy.

## Introduction

Ulcerative colitis (UC), referred to as one of the inflammatory bowel disease, is a kind of chronic recurrent diseases characterized by shallow ulcers in the mucosa of rectum and colon 1. The clinical manifestations of UC in the acute phase are abdominal pain, as well as diarrhea with mucus or blood, and long-term illness contributes to the occurrence of colorectal cancer 2. At present, the pathogenesis of UC still remains to be further elucidated, and there are certain limitations in the therapeutic strategy. Therefore, it is urgent to develop optimal drugs.

Multiple factors including heredity, environment, microbiota and mucosal immune regulation disorders contribute to UC 3. More and more evidence showed that intestinal microbiota imbalance and mucosal barrier dysfunction are closely related to UC occurrence 4, 5. The intestinal mucosal barrier consists of four parts including biological barrier, chemical barrier, epithelial barrier and immune barrier, which jointly prevent the intestinal wall from being invaded by bacteria and harmful substances 5. The damage of intestinal mucosal barrier is one of the most important characteristics in UC 6. Disordered microbiota could lead to the imbalance of the immune regulation in the intestine, affecting the integrity and repair of intestinal epithelial cells, and causing the exacerbation of intestinal inflammation 7, 8.

The abnormal activation of innate and adaptive immunity is a hallmark for the pathological process in UC. Activated immune cells will proliferate and release a series of effectors including TNF-α, IL-17A as well as other pathogenic factors, which further destroy the integrity of intestinal mucosal barrier and lead to the deterioration of UC. While, some immune cells, such as Tregs releasing protective cytokine IL-10, exert an anti-inflammatory effect 9. Experiments have shown that IL-22 is involved in the repair of epithelial cells during intestinal inflammation, and the main source of IL-22 is type 3 innate lymphoid cells (ILC3) in intestine 10, 11.

Berberine (BBR) is an important isoquinoline alkaloid extracted from Chinese medicine such as Coptischinensis and Phellodendronchinense. It exhibits great anti-bacterial and anti-diarrhea effects with few side effects 12. Previous studies have shown that BBR can relieve acute and chronic experimental colitis by regulating Th17/Treg balance 12, intestinal microbiota balance and metabolism 14, as well as intestinal glial-epithelial-immune cell interaction 15. However, the further mechanism of BBR in alleviating UC still needs to be clarified.

In this study, we explored the mechanism of BBR in treating UC, and systematically investigated the regulation of BBR on intestinal mucosal barrier and immune homeostasis in the colon. Finally, we analyzed and validated the signaling pathway relating to therapeutic effect of BBR on UC using proteomics and inhibitor intervention.

## Materials and methods

### Experimental animals

6-week-old male Balb/c mice were used in this study. All mice were purchased from Beijing Vital River Laboratory Animal. Technology Co., Ltd. and housed in the breeding room with specific pathogens free (SPF) in the animal center of Tongji Medical College, Huazhong University of Science and Technology (HUST). During the experimental period, the mice were housed in a standard environment (temperature 24±2℃, humidity 55±10%, and 12-hour day/night cycle) with free access to food and water. All animal experiments were performed strictly according to the Animal Research Institute Committee guidelines which were approved by the Institutional Animal Care and Use Committee (IACUC) of HUST.

### Establishment of UC model and administration of drugs

All mice were fed adaptively for 5 days before experiment. 3% (w/v) dextran sodium sulfate (DSS, 36-50 kDa, MP Biomedicals, Cat# 160110) dissolved in drinking water was used to induce the acute colitis model for 7 days according to the description of Wirtz et al 16. After starting the model induction, the administrated mice were given BBR (Shanghai yuanye Bio-Technology Co., Ltd, Cat# 2086-83-1) in a gavaged dose of 40 mg/kg per day according to the previous study 13, and other mice were gavaged with an equal volume of distilled water. The inhibitor of Wnt pathway was administered daily via intraperitoneal injection since model induction according to the manufacturer's instruction, while the solvent of inhibitor was injected with the same method and volume to eliminate the possible interference of the solvent.

### Assessment of UC and sample collection

Body weight, stool consistency and stool bleeding, were recorded daily for colitis severity indication. The disease activity index (DAI) score was calculated as described previously 17. After the model induction was completed, the mice were anesthetized by pentobarbital sodium and sacrificed through cervical dislocation. The colon was isolated and its length was measured. Colonic tissue at the distance of 1 cm from the anus was excised, rinsed, and placed in 4% formalin for pathological analysis. The remaining colon was open longitudinally, and the feces were rinsed gently with pre-cooled phosphate-buffered saline (PBS) for further research.

### Microbiota depletion and fecal microbiota transplantation (FMT)

Antibiotic cocktails including vancomycin (100 mg/kg, Macklin, Cat# V871983), neomycin sulfate (200 mg/kg, Cat# N6063), metronidazole (200 mg/kg, Cat# M813526) and ampicillin (200 mg/kg, Cat# A830931) were used for microbiota depletion for consecutive 2 weeks^13^. FMT was conducted according to previous description with minor revision 13. Briefly, fresh feces on the 7^th^ day after model induction of donor mice (DSS group and DSS+BBR+ group) were collected, homogenized with 0.1 g/ml saline solution and filtered. The recipient mice were gavaged with 0.2 ml of fresh solution once per day for 7 consecutive days after model induction.

### Proteomic analysis

150mg colon tissue per sample was used for protein lysis and extraction with SDT buffer (4% SDS, 100mM Tris-HCl, 1mM DTT, pH 7.6). The extracted protein was processed with specific buffers to prepare filter-aided sample (FASP Digestion) and obtain protein peptide. Appropriate amount of peptide mixture for each sample were taken and labeled with TMT reagent according to the manufacturer's instructions, and the labeled peptides were fractionated by high pH Reversed-Phase Peptide Fractionation Kit (Thermo Scientific). Next, the purified peptide fragments were analyzed on a Q Exactive mass spectrometer (LC-MS/MS, Thermo Scientific) for 69/90 min, and the mass spectrometer was operated with the peptide recognition mode. The obtained data was identified and quantitatively analyzed using Proteome Discoverer 1.4 software. Finally, bioinformatics analysis was performed.

### Intestinal permeability detection

FITC-dextran (4kDa, Sigma, Cat# 68059) was prepared into a solution under dark condition. After fasting for 10 hours, the mice were gavaged with the solution at the dose of 0.5 g/kg of FITC-dextran. After keeping in the dark for 2 hours, mouse blood was taken from the eye socket and placed in an EP tube containing anticoagulant. Subsequently, all mice were killed as described above, the liver and mesenteric lymph nodes (MLNs) were isolated and homogenized with PBS. Peripheral blood and homogenized tissues were centrifuged, and the supernatant was taken. After dilution, the OD values were measured using a fluorescence microplate reader, and the standard curve was obtained according to the concentration gradient of FITC-dextran to calculate and the fluorescence intensity.

### Histopathological evaluation

Mice colonic tissue were embedded in paraffin and sectioned after fixing in paraformaldehyde for 24 hours. Hematoxylin-eosin (HE) staining was used for histological analysis, while periodic acid-schiff (PAS) staining for assessing the number of goblet cells, and alcian blue staining for evaluating the amount of mucus secretion. All histological images were captured through panoramic scanner. Inflammation was scored blindly using the scoring system described by Kihara et al 18. Simply, the four parameters including inflammation severity, inflammation extent, crypt damage and percent involvement are used to determine the score. Each parameter is graded from 0-3 according to the severity of colonic changes. A higher score means that the inflammation is severer in colon.

### Fecal DNA extraction and microbial identification

During the model induction, 100 mg of feces were collected in a sterile EP tube from each mouse every day and quickly store at -80℃. E.Z.N.A.^®^Stool DNA Kit (Omega, Cat# D4015-01) was used to extract fecal DNA according to the manufacturer's protocol. After DNA extraction, the relative expressions of total fecal bacteria amount and specific bacteria were detected by PR-PCR. The primer sequences are shown in **Table 1**.

### Cell culture and inflammatory induction

The Caco2 cell line was purchased from American Type Culture Collection (ATCC). DMEM medium (Gibco, Cat# C11995500CP) containing 10% Fetal Bovine Serum (FBS, Gibco, Cat# 10099141), 100U/ml penicillin and 100μg/ml streptomycin (HyClone, Cat# SV30010) was used for Caco2 culture at the condition of 37℃ with 5% CO_2_. When the cells adhere to the wall of culture flask and grow to 70-80% confluence, trypsin (Gibco, Cat# BL512A) was used to digest the cells for cell passage. The medium was replaced regularly according to the cell growth, and the tight junction was formed after cell fusion, which means the cells can be used for related experimental research. The cell model was induced with 50ng/ml of TNF-α (Peprotech, Cat# AF-300-01A) for 24h as described previously^19^, and 20μM of BBR was pre-treated before the model induction for 2h according to our preliminary experiment.

### Fecal supernatant preparation and intervention

Mouse feces were collected in a pollution-free environment, and sterile PBS was added at a ratio of 100 mg/ml and vortexed repeatedly to make it dispersed. Then the suspension was added with 1640 medium (Gibco, Cat# C11875500CP) at a ratio of 1:8, incubated at 37°C for 2h and centrifuged. The supernatant was taken, sterilized by filtration, stored at 4°C, and used within 48h. Before the supernatant intervention, the cells were pretreated with basic DMEM medium for 4h, and then treated with complete medium containing a certain concentration of supernatant but no Penicillin-Streptomycin for 12h, and relevant tests were performed.

### siRNA transfection

hWnt1 siRNA was used to transfect Caco2 cells. The primer sequences were 5'-CCUCUUCGGCAAGAUCGUCAATT-3' and 5'-UUGACGAUCUUGCCGAAGAGGTT-3'. 70%-90% confluent cells were used for transfection. Dilute siRNA and PEI with a certain volume of Opti-MEM Medium respectively, and then add siRNA to PEI followed by incubation for 15 min. The mixture was then added to the cells. After 24 hours, the medium was changed, and drug intervention was given at the corresponding time. Cells were harvested at 72 hours after transfection for subsequent testing.

### Real-time polymerase chain reaction (RT-PCR)

Total RNA was extracted from colonic tissue using RNAiso Plus (TaKaRa, Cat# 9109) as described previously 20. After RNA extraction, PrimeScript RT Master Mix (TaKaRa, Cat# RR036A) was used to generate the complementary DNA (cDNAs) of mRNAs. Then, the cDNAs were analyzed to confirm the changes of gene expression using SYBR Premix Ex Taq (TaKaRa, Cat# RR420A). And the highly expressed endogenous β-actin was used to normalize the expression of target gene in tissues. After amplification, the Ct values were obtained, and the method of 2^-ΔΔCt^ was used to calculate the relative expression of each target gene. The primer sequences are listed in **Table 1**.

### Cytokine detection

LEGENDplex^TM^&ELISA Kits (BioLegend) were used to detect the expression of multiple cytokines in the colonic tissue. Normal saline supplemented with a protease inhibitor cocktail was used to prepare the colonic tissue homogenate. After centrifugation, the supernatants were used to detecting the concentration of cytokines according the manufactures' protocol. And the flow cytometry was used to quantify the data.

### Western blot

RIPA lysis buffer supplemented with a protease inhibitor cocktail was used to extract proteins from colonic tissue. After the tissue homogenate and centrifugation, the lysate was collected and the protein concentration was detected using the BCA kit (Beyotime, Cat# P0010). The samples were added with loading buffer after adjusting the protein concentration and heated in metal bath at 95℃ for 5 minutes. An equal amount of protein sample was taken for SDS-Page gel electrophoresis, and the proteins were separated under appropriate electrotransfer conditions. The proteins were transferred to polyvinylidenefluoride (PVDF) membranes (Immobilon-P, Cat# IPVH00010), which were then blocked in 5% skim milk (BD, Cat# 232100) for 1 h at room temperature (RT). The membranes were washed with TBST buffer, and then incubated with the appropriate primary antibody overnight at 4℃. After washing in TBST buffer for three times, the membrane was incubated with the corresponding secondary antibody at RT for 1 h and washed again, the membrane was exposed with Chemiluminescence Imaging System and quantitatively analyzed by imageJ software.

### Isolation of lamina propria cells in the colon and flow cytometry

The colon was open longitudinally and gently washed with pre-cooled PBS, then cut into 0.5 cm long pieces. First, Hank's buffer containing 5 mM EDTA (Gibco, Cat# AM9260G) and 10 mM Hepes (Gibco, Cat# 15630-080) was used to remove the epithelial cells for 20min at 37°C with shaking. After washing with PBS, the colonic tissue was digested with RPMI medium containing collagenase D (0.250 mg/ml) (Roche, Cat# 11088866001) and deoxyribonuclease (DNase) I (30 U/ml) (Roche, Cat# 10104159001) under the same condition for 20min. An appropriate amount of RPMI 1640 buffer containing 10% FBS was added to stop the digestion reaction, and the remaining colonic tissue were gently ground and filtered with a 100μm cell strainer to collect the solution. After centrifugation, the precipitate was collected. A 40/80% Percoll gradient was used to separate the cells at 800g for 20 minutes with a constant speed of up and down. The lamina propria cells in the middle layer were gently sucked out, and washed with FACS buffer twice. After centrifuging, the cells were re-suspended in FACS buffer to obtain a single cell suspension.

### Flow Cytometry

First, the cells isolated above were stained with surface markers, and lymphocytes were labeled with anti-CD45 antibody. Anti-CD4 antibody was used to identify T cell subset, and Lineage and anti-CD127 antibodies were used for ILCs identification, the lineage cocktail contained antibodies including NK1.1, CD11b, TER-119, Ly6G/Ly6C, CD11c, CD45R and CD3. Meanwhile, Fixable Viability Stain (BioLegend) was used to remove dead cells. All surface markers were stained in the dark at 4℃for 30 minutes. Next, the surface-stained cells were performed with the fixation and permeabilization buffer (Thermo Fisher Scientific, Cat# 88-8824-00) according to the manufacturer's protocol. Then, nuclear staining was performed with anti-T-bet and ROR-γt for ILCs and anti-ROR-γt and Foxp3 for T cell subset for 1 hours at 4℃. After washing, the cells were resuspended in FACS buffer and tested by FCM. Antibodies used for flow cytometry included anti-mouse CD45-FITC (BD, Clone: 30-F11), CD4-PE/Cy7 (BD, Clone: RM4-5), CD127-PE/Cy7 (BD, Clone: SB/199), ROR-γt-PE (BD, Clone: Q31-378), Foxp3-PerCP-Cy5.5 (BD, Clone: R16-715), T-bet-APC (BD, Clone: 4B10), Fixable Viability Stain 510 (BD, Cat# 564406), NK1.1-eFluor 450 (eBioscience, Clone: PK136), CD11b-eFluor 450 (eBioscience, Clone: M1/70), TER-119-eFluor 450 (eBioscience, Clone: TER-119), Ly6G/Ly6C-eFluor 450 (eBioscience, Clone: RB6-BC5), CD11c-eFluor 450 (eBioscience, Clone: N418), CD45R-eFluor 450 (eBioscience, Clone: RA3-6B2) and CD3-eFluor 450 (eBioscience, Clone: 17A2). Flow cytometry analysis was performed using CytExpert software and the gating strategies for each cell population were showed in **Figure S1**.

### Statistical analysis

All data are expressed as mean ± standard deviation (SD). The significant differences were determined using one-way ANOVAs followed by Dunnett's test in SPSS 22.0 software; p <0.05 was considered as a statistically significant. All proteomics sequencing results are analyzed on the cloud platform.

## Results

### Berberine relieves mouse colitis symptoms induced by DSS

**Figure 1:**
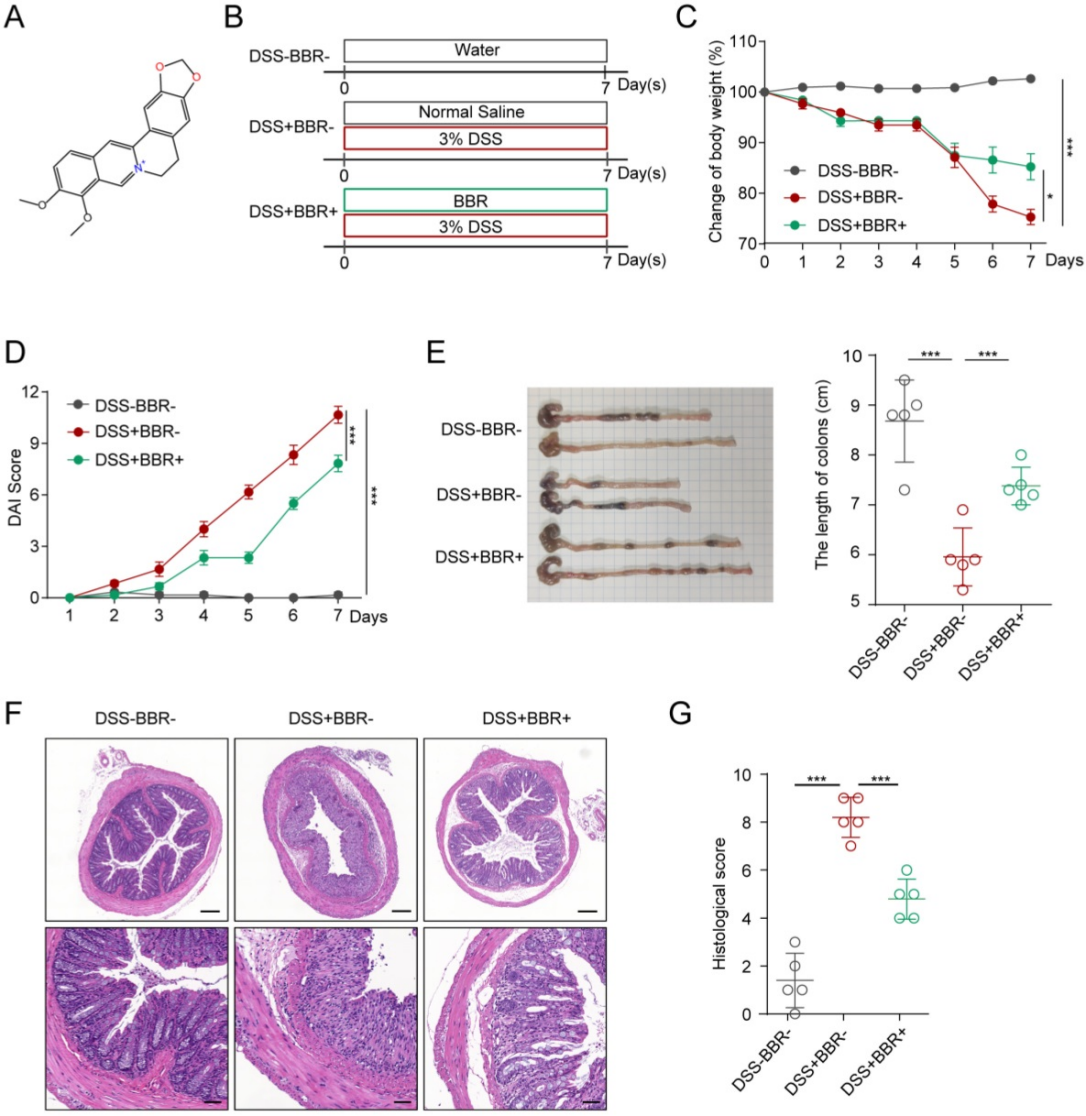
** BBR relieved DSS-induced colitis in mice.** (A) The molecular structure of BBR. (B) Schematic diagram illustrates the experimental design. (C) Body weight percentage changes of each group. (D) The effect of BBR on DAI in mice. (E) Measurement of the length of colons harvested from mice in each group. (F) H&E staining (Bar=200um above, Bar=50um below) sections and histological scores of colon tissue from mice in each group. Data are presented as mean ± *SD.* *P< 0.05, significantly different as indicated.

The molecular formula of BBR is C_17_H_17_N, and its structure is shown in Fig. 1A. In order to study the therapeutic effect of BBR on UC, we used DSS to induce an acute mouse colitis model (Fig. 1B). During the construction of the model, the mice experienced weight loss, progressively increased diarrhea with bloody and mucus stools. As shown in Fig. 1C, compared with the mice in DSS group, BBR significantly improved DSS-induced colitis with less weight loss. Similarly, it can significantly reduce the DAI score which reflects the severity of experimental colitis (Fig. 1D). Colon shortening is an important characteristic of DSS-induced colitis, and BBR significantly reversed the change (Fig. 1E). In addition, the DSS group had disordered glandular structure, reduced goblet cells, and a large number of inflammatory cell infiltration viewed by histopathological section staining, while BBR could alleviate these changes and reduce histopathological scores (Fig. 1F).

### Berberine ameliorates DSS-induced colitis in a microbiota-dependent manner

**Figure 2:**
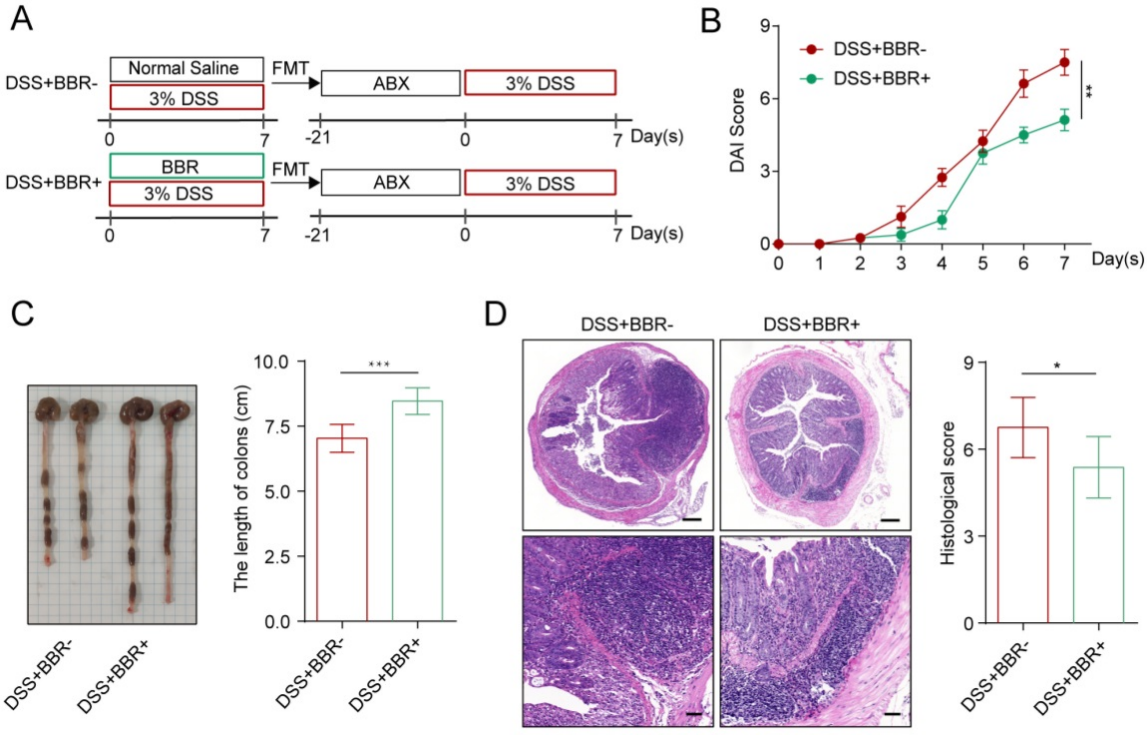
** BBR attenuated DSS-induced colitis in a microbiota-dependent manner.** (A) Schematic diagram illustrates the experimental design. (B) DAI changes of each group. (C) Measurement of the length of colons harvested from mice in each group. (D) H&E staining (Bar=200um above, Bar=50um below) sections and histological scores of colon tissue from mice in each group. Data are presented as mean ± *SD.* *P< 0.05, significantly different as indicated.

Intestinal microbiota disorder is closely related to the incidence of UC 4. Studies have shown that BBR can increase the total intestinal microbiota and the abundance of probiotics 13. Therefore, we further explored whether the therapeutic effect of BBR on DSS-induced colitis in mice depends on the microbiota. Two weeks before colitis induction in mice, ABX was used to deplete the intestinal microbiota 13. After model induction and drug intervention, we found that the microbiota depletion by ABX significantly reduced the therapeutic effect of BBR (Fig. S2A-D). Interestingly, ABX+DSS+-treated mice showed a better curative effect in DAI score, colon length, and histopathology after receiving fecal from BBR-treated colitis mice than those from UC model mice (Fig. 2A-D). These data indicate that the protective effect of BBR on DSS-induced colitis depends on intestinal microbiota.

### Berberine restores the biological and chemical barriers of colitis mice

**Figure 3:**
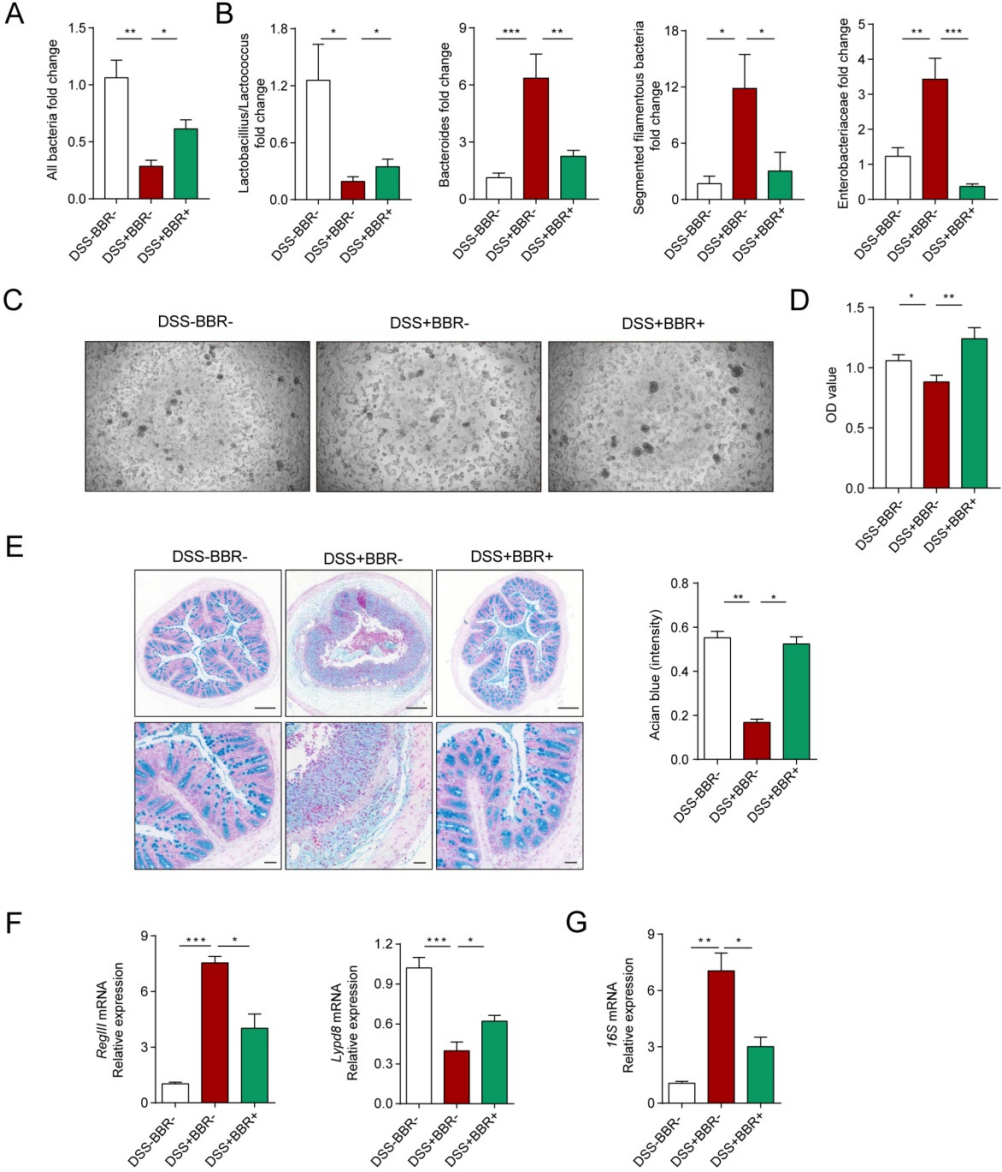
** Berberine restores the biological and chemical barriers of colitis mice.** (A) BBR treatment altered the total DNA of gut microbiota in fecal samples of colitis mice. (B) BBR treatment altered the structure of gut microbiota. (C) Represent images of Caco2 cells after the treatment of fecal supernatant from each group. (D) The effects of fecal supernatants on Caco2 cells viability. (E) Alcian blue staining (Bar=200um above, Bar=50um below) sections of colon tissue from mice in each group. (F) The expression levels of *RegIII* and *Lypd8* of colon in each group. (G) The expression of *16S mRNA* from microbiota attached to intestinal wall in each group. Data are presented as mean ± *SD.* *P< 0.05, significantly different as indicated.

The intestinal microbiota and thick mucus layer secreted by goblet cells covering the colonic epithelium constitute the biological and chemical barriers, respectively. In an inflammatory state, homeostasis of the microbiota is unbalanced, with the decrease of flora abundance as well as probiotics and increase of harmful bacteria 21. Meanwhile, the goblet cells decrease accompanied with the reduction of mucus secretion 22. In order to verify the protective effect of BBR on the biological barrier of DSS-induced colitis, we collected mouse feces and identified the changes among groups. 16S rRNA determines the total amount of bacteria, as shown in Fig. 3A, in model mice, the total amount of intestinal bacteria decreased significantly, while BBR can increase the relative level of bacteria. At the same time, compared with the model group, the expression of probiotics *Lactobacillius/Lactococcus* was up-regulated, but the expression of harmful bacteria including* mouse intestinal Bacteroides* (MIB), *segmented filamentous bacteria* (SFB) as well as *Enterobacteriaceae* were decreased (Fig. 3B). To identify the damage of bacteria on cells, the feces supernatants were prepared to intervene Caco2 cells. After 12-hours incubation, fecal supernatant from DSS group displayed the enormous cell-killing effect with rounded and shedding cells (Fig. 3C), which was verified by cell viability detection (Fig. 3D).

PAS staining was used to identify goblet cells and alcian blue staining for secreted mucus. As expected, DSS can significantly reduce the amount of goblet cells and mucus secretion, but BBR can improve the cells from exhaustion and mucus secretion (Fig. S3A, Fig. 3E). The RegIII protein derived from Paneth cells and the Lypd8 protein which is specifically expressed by colonic epithelial cells are essential for maintaining the intestinal bacteria separating away from epithelium 23, 24. And RegIII expression is related to the degree of inflammation, while the lypd8 is the opposite. The results display that BBR significantly restrained the increase of RegIII and decline of Lypd8 in mRNA level caused by DSS (Fig. 3F). Meanwhile, the thinned and destroyed mucus layer signifies an increase in bacteria located close to intestinal epithelium 25. Next, we analyzed the changes in adherent microbiota in isolated washed colons, and PCR analysis revealed an obvious up-regulation of 16s rRNA in DSS group compared to control and BBR groups (Fig. 3G). These data indicate that BBR was able to restore the damages of colonic biological and chemical barriers caused by DSS.

### Berberine repairs the integrity of the intestinal epithelial barrier

**Figure 4:**
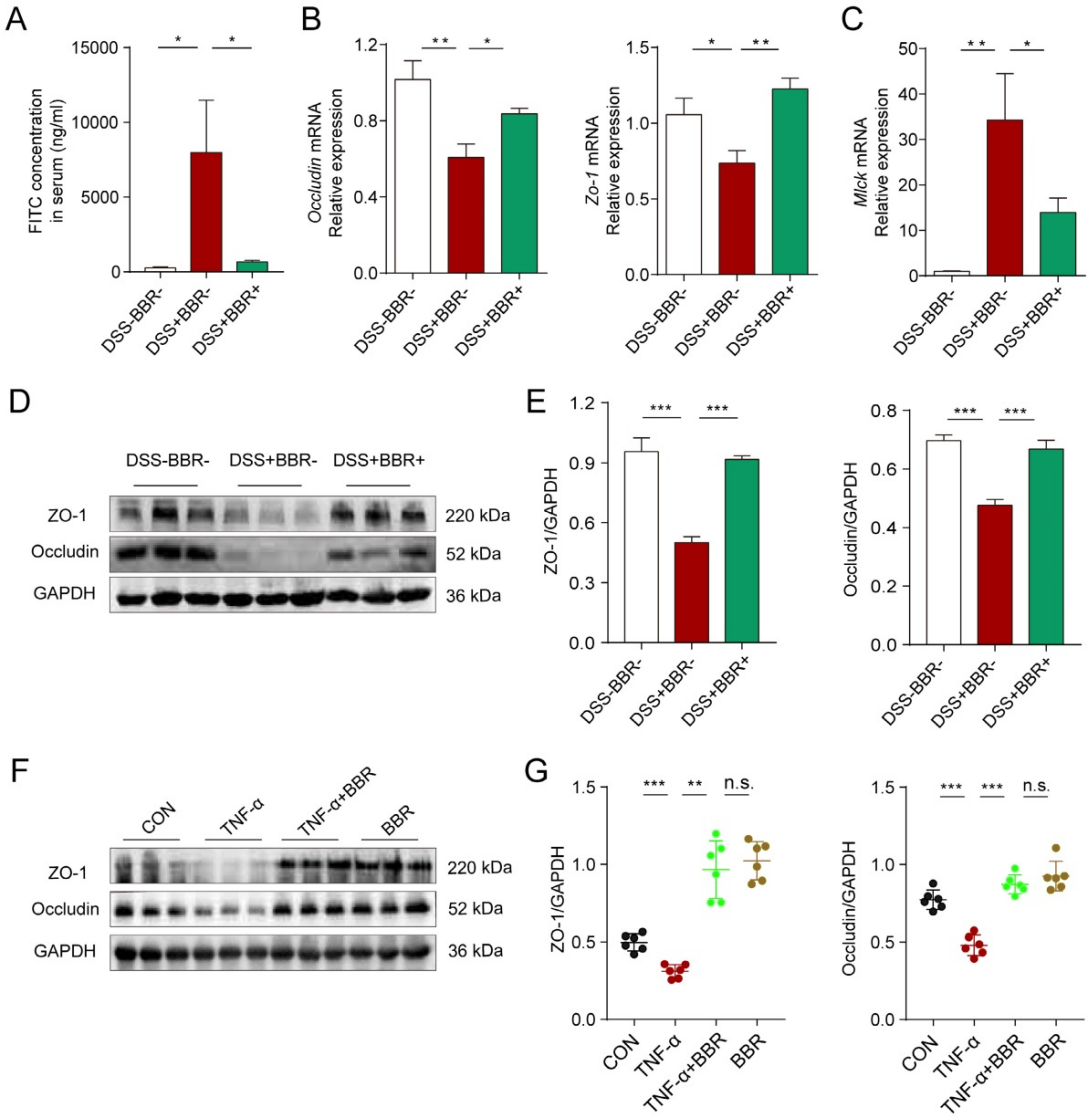
** BBR repaired the integrity of intestinal epithelial barrier in colitis mice.** (A) The serum FITC-dextran concentrations of mice in each group.(B) The colonic mRNA levels of *Occludin* and *Zo-1* in each group were measured by qPCR. (C) The colonic mRNA levels of *Mlck* in each group were measured by qPCR. (D) The colonic protein levels of Occludin and ZO-1 in each group were measured by western blotting. (E) Grey values of *ZO-1* and *Occludin* in colon normalized to GAPDH. (F) The protein levels of Occludin and ZO-1 in Caco2 cells were measured by western blotting. (G) Grey values of ZO-1 and Occludin in Caco2 cells normalized to GAPDH. Data are presented as mean ± *SD.* *P< 0.05, significantly different as indicated.

Intestinal epithelium is an important part of the intestinal mucosal barrier. In order to verify the effect of BBR on intestinal epithelial barrier, we conducted a series of experiments *in vivo* and *in vitro*. The intestinal tight junctions (TJs) and related protein expression are always used to assess the epithelial barrier damages 26. As shown in Fig. 4A and Fig. S3B, the fluorescence intensity of FITC in the blood, mesenteric lymph nodes (MLNs) and liver of mice in DSS group increased significantly, which means the increased intestinal permeability, while BBR can dramatically reduce the intensity of FITC in tissues. As the responsive indicator of TJs destruction, the protein and mRNA expression levels of Occludin and zonula occludens-1 (ZO-1) in colonic tissue of the DSS group were significantly reduced, while BBR significantly inhibited the decline of these indexes (Fig. 4B, D, E, Fig. S3D), and same tendency were saw in the *in-vitro* study, meanwhile, BBR did not cause the damage to TJ-related proteins of normal cells (Fig. 4F, G). The down-regulation of the MUC2 gene exacerbates the damage of epithelial barrier, while BBR up-regulated the mRNA level of MUC2 (Fig. S3C). Increased expression of myosin light chain kinase (MLCK) also leads to the destruction of TJs 27, as is shown in Fig. 4C, the mRNA expression of MLCK in colonic tissue of DSS group increased while that of the BBR group decreased. In summary, BBR can alleviate intestinal permeability, increase the expression of TJ-related proteins to relieve intestinal inflammation.

### Berberine regulates innate and adaptive immune homeostasis of colonic tissue

**Figure 5:**
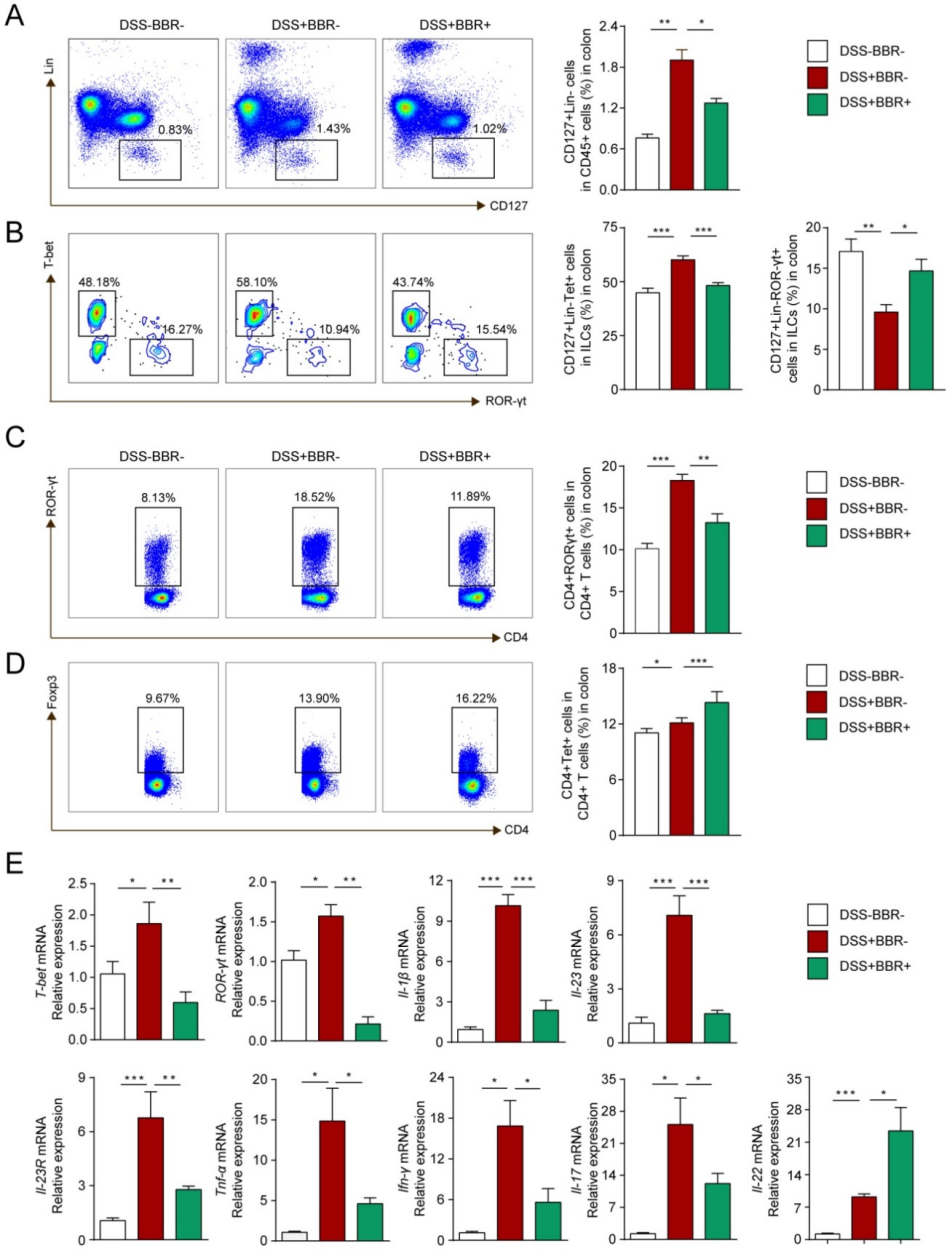
** BBR regulated innate and adaptive immune homeostasis of coloinc tissue.** (A) CD127^+^Lin^-^ cells (ILCs), (B) CD127^+^Lin^-^T-bet^+^ cell (ILC1) and CD127^+^Lin^-^ROR-γt^+^ cell (ILC3) in LPLs from each group were analyzed by flow cytometry. (C) ROR-γt^+^CD4^+^ (Th17) cells and (D) Foxp3^+^CD4^+^ (Treg) cells in LPLs from each group were analyzed by flow cytometry. (E) The colonic mRNA levels of *T-bet*, *ROR-γt* and inflammatory cytokines in each group were measured by qPCR. Data are presented as mean ± *SD.* *P< 0.05, significantly different as indicated.

ILCs play an important role in maintaining the integrity and function of the intestinal mucosal barrier in innate immunity, as well as the balance of Th17/Treg cells in adaptive immunity 28. To investigate the modulation of BBR on immune homeostasis, flow cytometry was used to analyze the phenotypes of colonic lamina propria lymphocyte (cLPL) in different groups. As shown in Fig. 5A and B, the percentages of total ILCs (Lin-CD127+) and ILC1 (Lin-CD127+T-bet+) in the DSS group increased, while ILC3 (Lin-CD127+ROR-γt+) decreased. After BBR administration, total ILCs and ILC1 decreased compared with the DSS group, and the percentage of ILC3 increased. Similarly, the changes of Th17 and Treg cells were consistent with other studies (Fig. 5C, D), namely that BBR could suppress the increase of Th17 cells in colonic tissue, and further increase the proportion of Tregs to restore the balance of Th17/Treg cells.

To further explore the effect of BBR on the differentiation and function of ILCs, we identified the expression of related transcription factors and cytokines in colonic tissue. Same to Th1 and Th17 cells, the differentiation of ILC1 and ILC3 is regulated by transcription factors T-bet and ROR-γt respectively and cytokines IL-1β, IL-23 as well as the receptor IL-23R 29. Other cytokines such as TNF-α, IFN-γ, IL-17 and IL-22 are secreted by ILC1 and ILC3 to exert pro-inflammatory or anti-inflammatory effects. As shown in Fig. 5E, With the intervention of BBR, compared with DSS group, the mRNA levels of pro-inflammatory indicators including T-bet, ROR-γt, IL-1β, IL-23, IL-23R, TNF-α, IFN-γ were decreased, but the expression of IL-22, a cytokine that promotes epithelial repair, was up-regulated. Meanwhile, the concentration of pro-inflammatory cytokines including IL-1β, TNF-α, IFN-γ and IL-17 as well as the anti-inflammtory cytokines IL-22 and IL-10 showed the same tendency with the results from qPCR (Fig. S4). In summary, our results indicated that BBR could regulate the intestinal immune responses.

### The effect of berberine on the alteration of proteomics in colon tissue

**Figure 6:**
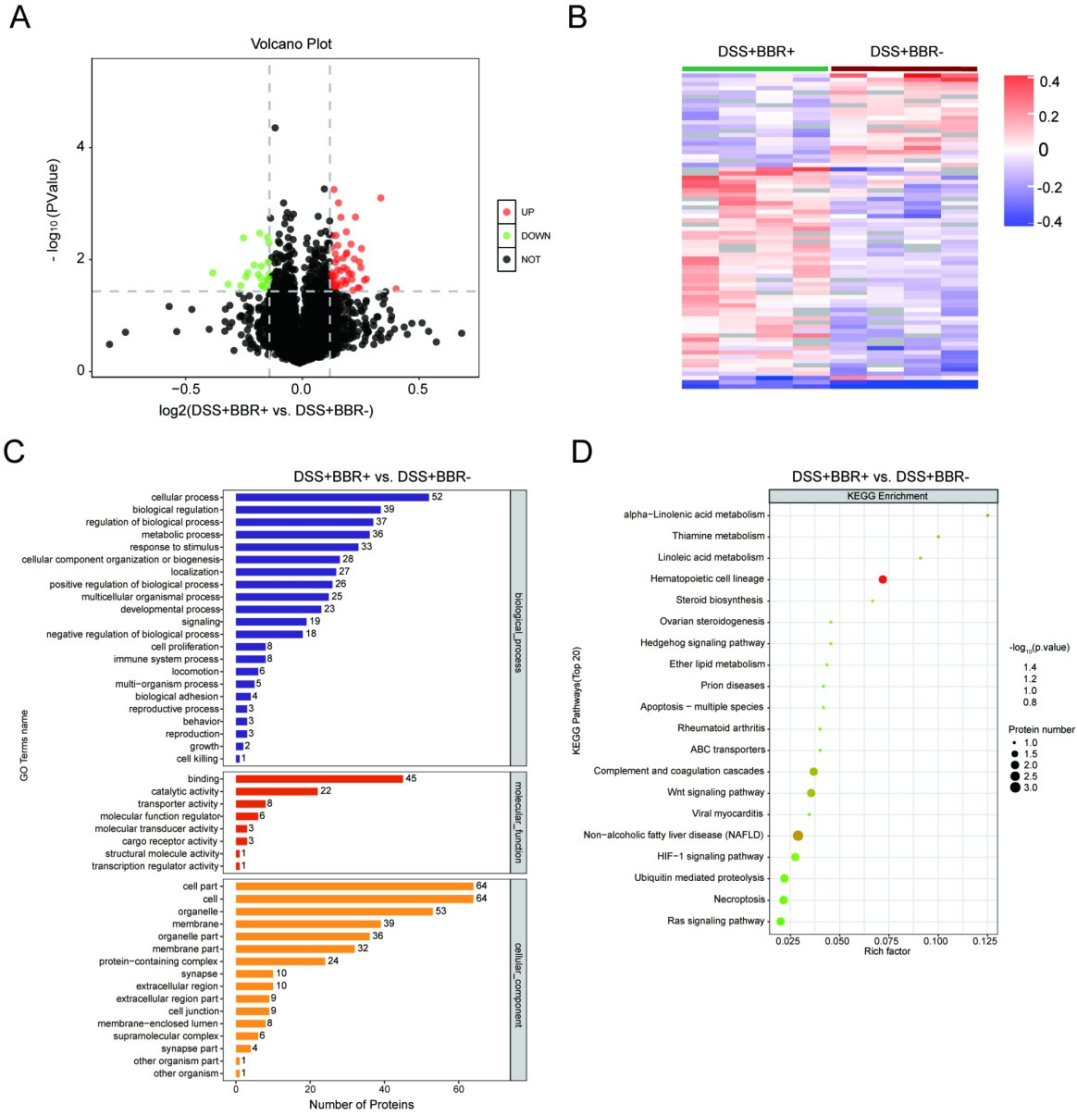
** BBR treatment significantlyalter the proteomics of colon tissue.** Volcano graph of the distribution of the different proteomics in DSS+BBR+ and DSS+BBR- groups, the red dots represents up-regulated proteins and green dots represent down-regulated proteins. (B) Hierarchical clustering of proteins in DSS+BBR+ and DSS+BBR- groups. (C) GO biological process analysis in DSS+BBR+ and DSS+BBR- groups. (D) KEGG pathways analysis in DSS+BBR+ and DSS+BBR- groups.

In order to understand the underlying pathogenesis of UC and the therapeutic target of BBR, we analyzed the proteomics of colonic tissues from three groups. The potential signaling pathways by which BBR works were screened in colonic tissues from mice with BBR administration. Compared with the control group, 92 proteins were up-regulated accompanied with 200 proteins down-regulated in DSS group (Fig. S5A); after BBR therapy, 51 proteins were up-regulated and 23 proteins were down-regulated compared to DSS group (Fig. 6A). Cluster analysis showed the limited differences within the groups and few similarities between two groups (Fig. S5B, Fig. 6B). Selected differentially expressed proteins and their expression frequencies are displayed between control and DSS groups, as well as DSS and BBR groups, according to GO biological process analysis, molecular function analysis, and cell component analysis (Fig. S5C, Fig. 6C). Next, we tried to determine the signaling pathways according to the differentially expressed proteins. The top 20 of KEGG pathways showed that complement and coagulation cascade, staphylococcus aureus infection, arginine and proline metabolism pathways were related to DSS-induced colitis (Fig. S5D), while Hematopoietic cell Lineage, Non-alcoholic fatty liver disease, and Wnt signaling pathway were closely associated with BBR therapy (Fig. 6D); it is worth noting that the complement and coagulation cascade played a critical role in both DSS-induced colitis and BBR administration, which is consistent with the previous research, that is, complement activation and the components of this pathway are involved in the colonic inflammatory response 30.

### Berberine improves DSS-induced colitis via Wnt/β-catenin pathway

**Figure 7:**
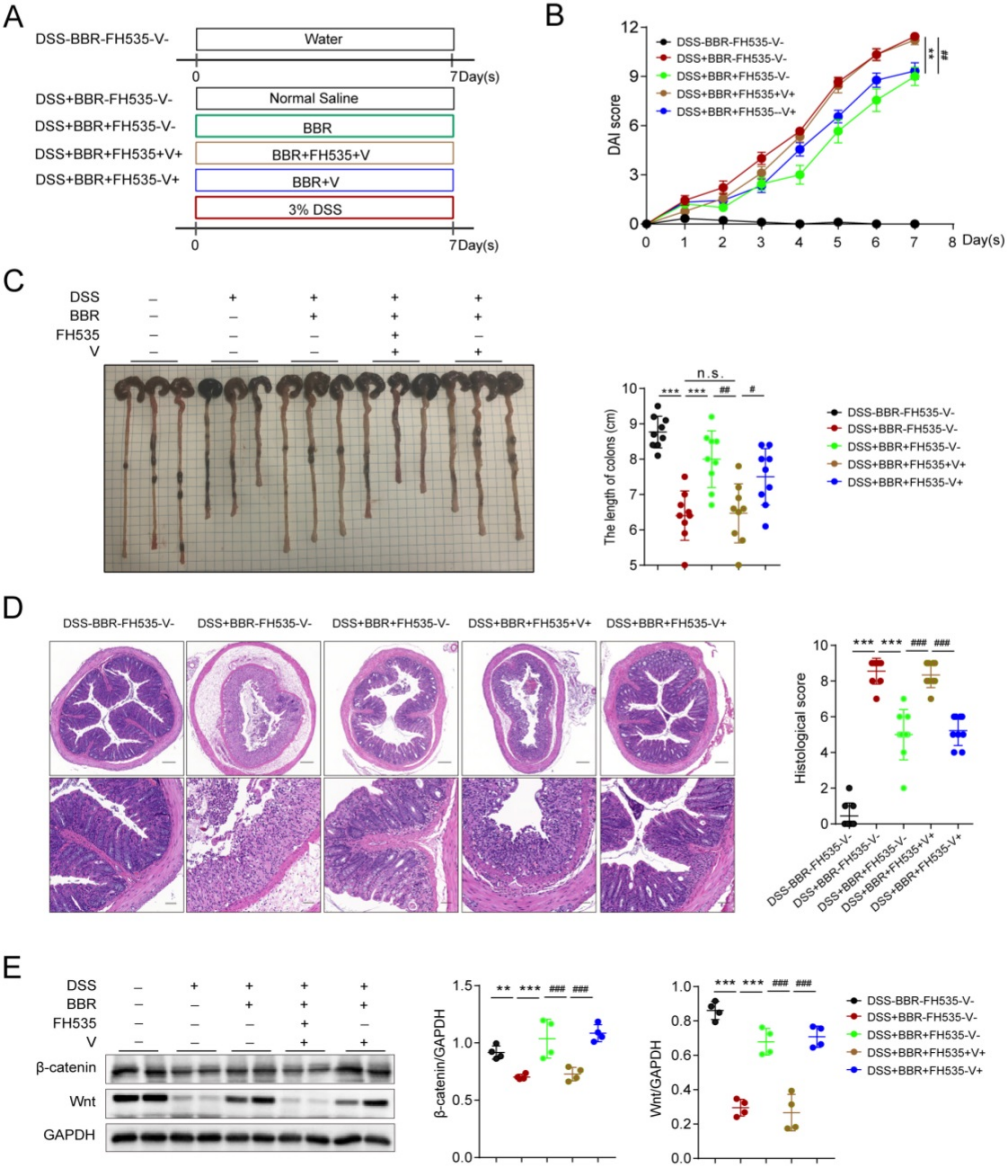
** BBR improves DSS-induced colitis via Wnt/β-catenin pathway.** Schematic diagram illustrates the experimental design. (B) DAI changes of each group. (C) Measurement of the length of colons harvested from mice in each group. (D) H&E staining (Bar=200um above, Bar=50um below) sections and histological scores of colon tissue from mice in each group. (E) The colonic protein levels of β-catenin and Wnt in each group were measured by western blotting. FH535 represents for the inhibitor of Wnt pathway and V for solvent of FH535. Data are presented as mean ± *SD.* *P< 0.05, significantly different as indicated.

Data from proteomics suggested that the therapeutic effect of BBR might be related to the Wnt pathway, which has been proved to exert great effect on the proliferation of epithelial cells in repairing mechanical barrier 31. The expression of β-catenin is regulated by Wnt, and they participate in the physiological and pathological process of injure 32. In order to confirm whether the therapeutic effect of BBR on UC is Wnt/β-catenin pathway dependent, we conducted the experiment with FH535 (MedChemExpress, Cat# HY-15721), the inhibitor of Wnt pathway (Fig. 7A). We found that Wnt/β-catenin pathway blockade eliminated the therapeutic effect of BBR on intestinal inflammation, which was reflected in the changes of DAI score, colon length and pathological damage, while the solvent of FH535 did not lead to the alternation (Fig. 7B-D). Moreover, compared with normal mice, the protein levels of Wnt and β-catenin were significantly decreased, and BBR was able to increase their expression. However, the promotion of BBR on the expression of Wnt and β-catenin were disappeared after administration of FH535 (Fig. 7E).

**Figure 8:**
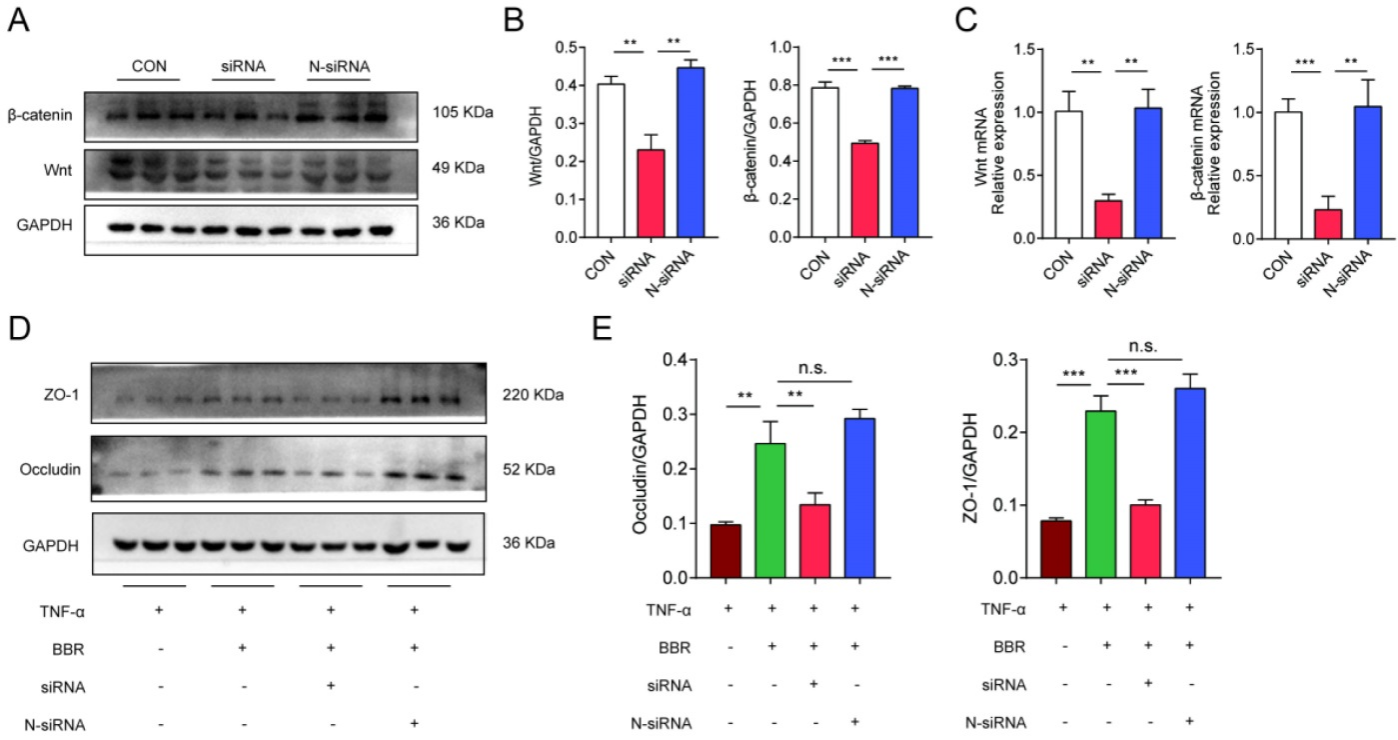
** BBR protects TNF-α induced Caco2 cell damages via Wnt/β-catenin pathway.** (A) The transfection efficiency of siRNA on the expression of Wnt and β-catenin was measured by western blotting. (B) Grey values of Wnt and β-catenin normalized to GAPDH. (C) The mRNA levels of Wnt and β-catenin in transfected cells measured by qPCR. (D) The protein levels of Occludin and ZO-1 in Caco2 cells with transfection were measured by western blotting. (E) Grey values of Occludin and ZO-1 normalized to GAPDH. Data are presented as mean ± *SD*. *P< 0.05, significantly different as indicated.

Meanwhile, we used Wnt1 siRNA to transfect Caco2 cells to further clarify the role of Wnt/β-catenin pathway in the colonic protection of BBR. We firstly clarified the silence efficiency of siRNA and siRNA-con on the expression of Wnt and β-catenin. siRNA was able to significantly reduce the mRNA and protein levels of Wnt and β-catenin in Caco2 cells (Fig. 8A-C), while N-siRNA had no such effect. The previous results have confirmed that BBR could protect TNF-α induced TJ damage in Caco2 cells, then, the siRNA and N-siRNA were used to intervene the cells treating with TNF-α and BBR to determine the role of Wnt/β-catenin pathway. Consistently, siRNA weakened the protective effect of BBR on TNF-α induced damage on Caco2 cells, which was demonstrated by the down-regulation of protein levels of Occludin and ZO-1 (Fig. 8D, E). Overall, these data indicated that the Wnt/β-catenin pathway was related to the pathogenesis of UC and BBR administration, and BBR exerted therapeutic role on DSS-induced colitis relying on the Wnt/β-catenin pathway.

## Discussion

Berberine has shown great ability in treating intestinal diseases, and it is often used clinically for diarrhea 33. For the past few years, researchers have shown great interest in the therapeutic effects and mechanisms of BBR on IBD. In several acute and chronic UC models, BBR is able to improve colonic morphology macroscopically and alleviate histopathological inflammation microscopically, and exert a significant anti-UC effect 13, 15, 34. In this study, we comprehensively and systematically elucidated the approaches by which BBR alleviates UC, the protective effect on the intestinal mucosal barrier, and the signaling pathways it relies on by preforming a series of experiments.

Studies have found that the compositions of gut microbiota of patients with UC are significantly different from those of healthy individuals 4. The decrease of microbiota abundance and disproportionality of probiotics and harmful bacteria play vital roles in the occurrence of UC 13. Meanwhile, microbiota alteration leads to the change of its derived metabolites, which affects intestinal health greatly 35. Therefore, it is of broad prospects for maintaining the homeostasis of gut microbiota in UC therapy. Accumulating evidences have shown that re-establish the balance of microorganism can alleviate the DSS-induced mouse colitis 13, 36. It is worth noting that the intestinal absorption of BBR is poor with oral administration, most of which still stay in the intestinal cavity and directly interact with microbes 37. Therefore, the gut microbiota may be an important target for BBR to exert therapeutic effects, and our experiments have just confirmed the point. After microbiota depletion, the therapeutic effect of BBR disappeared, while transplanting fecal from mice in the BBR group can improve the DSS-induced microbiota-depleted mouse colitis. Although the therapeutic effect caused by residual BBR in the stool cannot be ruled out, the results are sufficient to prove that BBR works in a microbiota-dependent manner. Meanwhile, our results showed that BBR is able to regulate microbiota abundance and composition, which demonstrated the regulation on biological barrier of mice with DSS-induced colitis.

The destroyed biological barrier aggravates intestinal inflammatory response, which affects the function of intestinal chemical barrier. Under physiological conditions, the goblet cells are abundant accompanied with normal secretion functions, and the mucus layer is rich in antimicrobial peptides, which effectively maintains the balance between intestinal bacteria and epithelial cells and prevents infection 22. Decreased goblet cells as well as antimicrobial peptides, damaged mucus layer, and increased mucus and epithelial attached bacteria are the pathological features of UC, which in turn amplifies the intestinal inflammation 22. Mice do show chemical barrier damage after induction by DSS, and our experiments have confirmed that the intervention of BBR can significantly inhibit the impairment of DSS on chemical barrier in model mice.

Furthermore, the epithelial barrier and immune barrier, as the first line of defense in the intestine, can maintain the integrity of the intestinal mucosa and effectively prevent harmful substances from invading the intestinal wall 38. A growing number of studies have shown that promoting repair of intestinal epithelial, increasing TJs expression, and regulating the balance of T cell subsets enable alleviation of intestinal inflammatory response^34^, ^39^, and our results showed that with administration of BBR in DSS-induced mouse colitis, the integrity of epithelial cells and Th17/Treg balance are improved. The restore of epithelial barrier is regulated by IL-22, whose main source in colonic tissue is ILC3 according to studies 34, 39. The subsets of ILCs are similar to those of Th cells, and they are highly expressed in intestinal mucosa, dynamically regulating tissue balance. It is worth noting that ILC1 and ILC3 are the main components of intestinal ILCs, while ILC2 is less expressed 41. Studies have shown that in patients with IBD, the proportion of ILC1 in colon is increased while ILC3 decreased, accompanied by alteration in the expression of their effector molecules 42. Surprisingly, BBR can inhibit the expression of ILC1 in the cLPLs of DSS-induced mice but up-regulate the percentage of ILC3 as well as the expression of IL-22. These data means that BBR is able to promote intestinal epithelial barrier repair, modulate colonic innate and adaptive immune response to exert therapeutic effects.

In the intestine, the activation of Wnt/β-catenin signaling pathway, which occupies a dominant position in the recognition and maintenance of epithelial stem cells, is essential for maintaining epithelial homeostasis 31. The Wnt/β-catenin signaling pathway is closely associated with other inflammatory signaling pathways including NF-κB and MAPK signals, and affects epithelial homeostasis as well as tissue regeneration 43. And the pathway inhibition leads to the loss of crypts and tissue denaturation 43. Furthermore, research has shown that the repair of intestinal mucosal barrier by anti-inflammatory factor IL-10 partly depends on the activation of Wnt pathway in epithelial cells 44. Through intestinal proteomics, we found that BBR can increase the expression of Wnt pathway in intestinal tissues. However, with the intervention of Wnt inhibitors, the therapeutic effect of BBR is significantly reduced, which demonstrates that the protective effect of BBR on UC is at least partly dependent on the Wnt/β-catenin signaling pathway.

**Figure 9:**
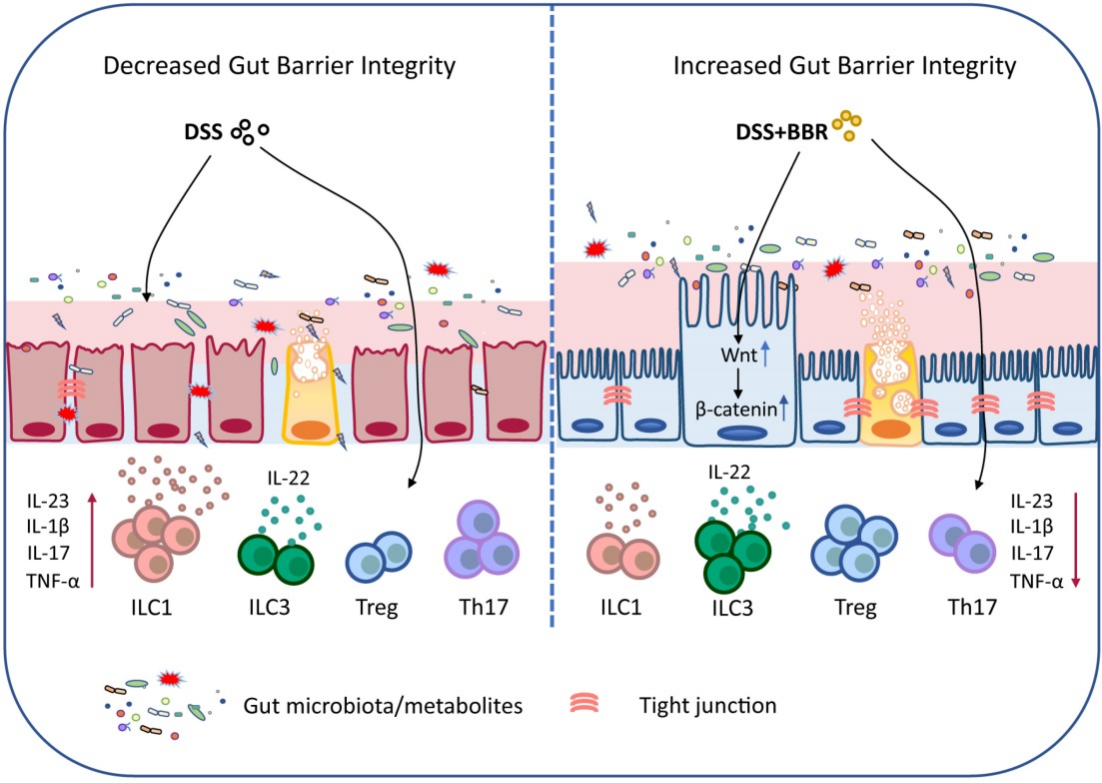
Schematic depiction about the protective effects of BBR during ulcerative colitis.

In summary, our data demonstrated that BBR plays a therapeutic role in regulating microbiota, maintaining the chemical and epithelial barriers, and regulating intestinal mucosal immune responses, and it works through the microbiota-dependent manner and Wnt/β-catenin signaling pathway (Fig. 9). Meanwhile, the results further prove that BBR may be a promising drug candidate for UC therapy. Well, the deeper mechanisms of the main bacterial species or metabolites that BBR relies on in the treatment of UC, and how to improve its effective utilization and expand the therapeutic effect will be our future research.

## Supplementary Material

Supplementary figures.

## Figures and Tables

**Table 1:** Primer sequences for RT-PCR.

Gene		Primer sequences (5'-3')
16S rRNA	Sense	ACTCCTACGGGAGGCAGCAGCAGT
	Anti-sense	TATTACCGCGGCTGCTGGC
Lactobacillius/Lactococcus	LabF362	AGCAGTAGGGAATCTTCCA
	LabR677	CACCGCTACACATGGAG
MIB	Uni516F	CCAGCAGCCGCGGTAATA
	MIBR677	CGCATTCCGCATACTTCTC
SFB	SFB736F	GACGCTGAGGCATGAGAGCAT
	SFB844R	GACGGCACGGATTGTTATTCA
Enterobacteriaceae	515F	GTGCCAGCMGCCGCGGTAA
	826R	GCCTCAAGGGCACAACCTCCAAG
M-β-actin	Sense	GTGACGTTGACATCCGTAAAGA
	Anti-sense	GTAACAGTCCGCCTAGAAGCAC
H-β-actin	Sense	CACCATTGGCAATGAGCGGTTC
	Anti-sense	AGGTCTTTGCGGATGTCCACGT
M-RegIII	Sense	CGTGCCTATGGCTCCTATTGCT
	Anti-sense	TTCAGCGCCACTGAGCACAGAC
M-Lypd8	Sense	AACACTCTGCGAGGAGAAACCC
	Anti-sense	AGAACAGCCCTTCAGCTCCACT
M-ZO-1	Sense	GTTGGTACGGTGCCCTGAAAGA
	Anti-sense	GCTGACAGGTAGGACAGACGAT
M-Occludin	Sense	TGGCAAGCGATCATACCCAGAG
	Anti-sense	CTGCCTGAAGTCATCCACACTC
M-MLCK	Sense	CACTGTCACCTGGTCGCTGAAT
	Anti-sense	GCGCTGTTCTTGGCTACACACT
H-ZO-1	Sense	GTCCAGAATCTCGGAAAAGTGCC
	Anti-sense	CTTTCAGCGCACCATACCAACC
H-Occludin	Sense	ATGGCAAAGTGAATGACAAGCGG
	Anti-sense	CTGTAACGAGGCTGCCTGAAGT
M-Muc2	Sense	GCTGACGAGTGGTTGGTGAATG
	Anti-sense	GATGAGGTGGCAGACAGGAGAC
M-IL-17	Sense	TCAGACTACCTCAACCGTTCCA
	Anti-sense	CAGCTTTCCCTCCGCATT
M-IL-22	Sense	GCTTGAGGTGTCCAACTTCCAG
	Anti-sense	ACTCCTCGGAACAGTTTCTCCC
M-IL-1β	Sense	TGGACCTTCCAGGATGAGGACA
	Anti-sense	GTTCATCTCGGAGCCTGTAGTG
M-IL-23	Sense	CATGCTAGCCTGGAACGCACAT
	Anti-sense	ACTGGCTGTTGTCCTTGAGTCC
M-IL-23R	Sense	GTCCACCAAACTTCCCAGACAG
	Anti-sense	CCTGAAGCAGGATGTCCTCTGA
M-TNF-α	Sense	GGTGCCTATGTCTCAGCCTCTT
	Anti-sense	GCCATAGAACTGATGAGAGGGAG
M-IFN-γ	Sense	CAGCAACAGCAAGGCGAAAAAGG
	Anti-sense	TTTCCGCTTCCTGAGGCTGGAT
M-ROR-γt	Sense	TCAGGAGTGCTTACTGTCGGTC
	Anti-sense	AGTTCTTCGGGGCTGGAAT
M-T-bet	Sense	CCACCTGTTGTGGTCCAAGTTC
	Anti-sense	CCACAAACATCCTGTAATGGCTTG

## Abbreviations

UC: ulcerative colitis
BBR: berberine
ILC: innate lymphoid cell
DSS: dextran sodium sulfate
DAI: disease activity index
PBS: phosphate-buffered saline
FMT: microbiota transplantation
PAS: periodic acid-schiff
MIB: mouse intestinal Bacteroides
SFB: segmented filamentous bacteria
TJs: tight junctions
ZO-1: zonula occludens-1
MLCK: myosin light chain kinase
