# Supplementary material for: Berberine ameliorates DSS-induced intestinal mucosal barrier dysfunction through microbiota-dependence and Wnt/β-catenin pathway
Source: Int J Biol Sci. 2022 Jan 16;18(4):1381–97. doi: 10.7150/ijbs.65476 (PMC8898376; doi:10.7150/ijbs.65476)
Supplement: Supplementary file 1 — Supplementary figures. [file ijbsv18p1381s1.pdf]

Figure S1. Flow gating strategies for each cell population.

Figure S2. Effects of BBR against DSS-induced colitis after pre-treatment with ABX.

(A) Schematic diagram illustrates the experimental design. (B) DAI changes of each group. (C) Measurement of the length of colons harvested from mice in each group. (D) H&E staining (Bar=200um above, Bar=50um below) sections and histological scores of colon tissue from mice in each group. Data are presented as mean ± *SD*. **P*< 0.05, significantly different as indicated.

Figure S3. BBR repaired the integrity of intestinal epithelial barrier in colitis mice.

(A) Paneth staining(Bar=200um above, Bar=50um below) sections of colon tissue from mice in each group. (B) The MLNs and liver FITC-dextran concentrations of mice in each group. (C) The colonic mRNA level of *Muc2* in each group were measured by qPCR. (D) The mRNA levels of *Occludin* and *Zo-1* in Caco2 cells from each group were measured by qPCR. Data are presented as mean ± *SD*. **P*< 0.05, significantly different as indicated.

Figure S4. BBR regulates the expression of cytokines in the colon.

(A) The concentration of inflammatory cytokines in each group were measured by ELISA. Data are presented as mean ± SD. **P*< *0.05*, significantly different as indicated.

Figure S5. BBR treatment significantly alter the proteomics of colon tissue.

(A) Volcano graph of the distribution of the different protein in DSS-BBR- and DSS+BBR- groups, the red dots represents up-regulated proteins and green dots represent down-regulated proteins. (B) Hierarchical clustering of proteins in DSS-BBR- and DSS+BBR- groups. (C) GO biological process analysisinDSS-BBR- and DSS+BBR- groups. (D) KEGG pathways analysis in DSS-BBR- and DSS+BBR- groups.

Figure S1

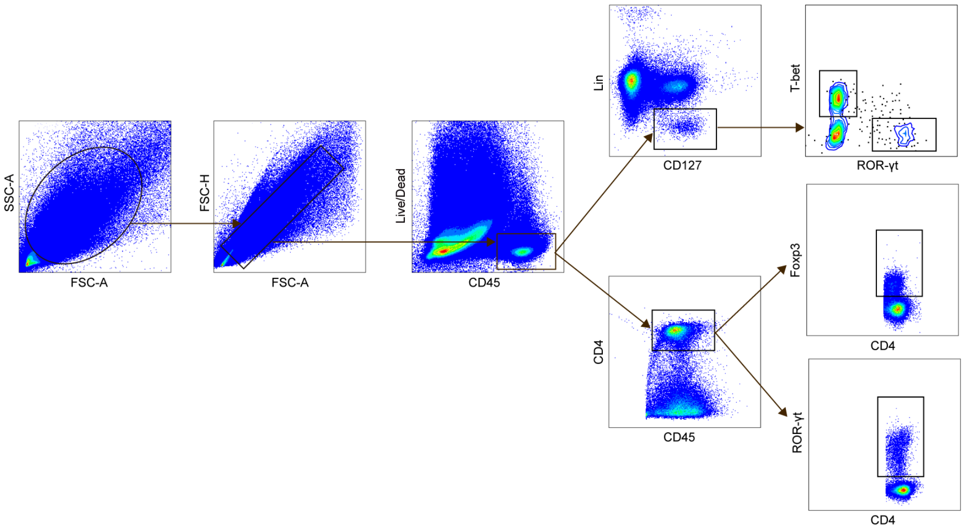
SSC-A
FSC-A
FSC-H
FSC-A
Live/Dead
CD45
Lin
CD127
T-bet
ROR-γt
CD4
CD45
Foxp3
CD4
ROR-γt
CD4

Figure S2

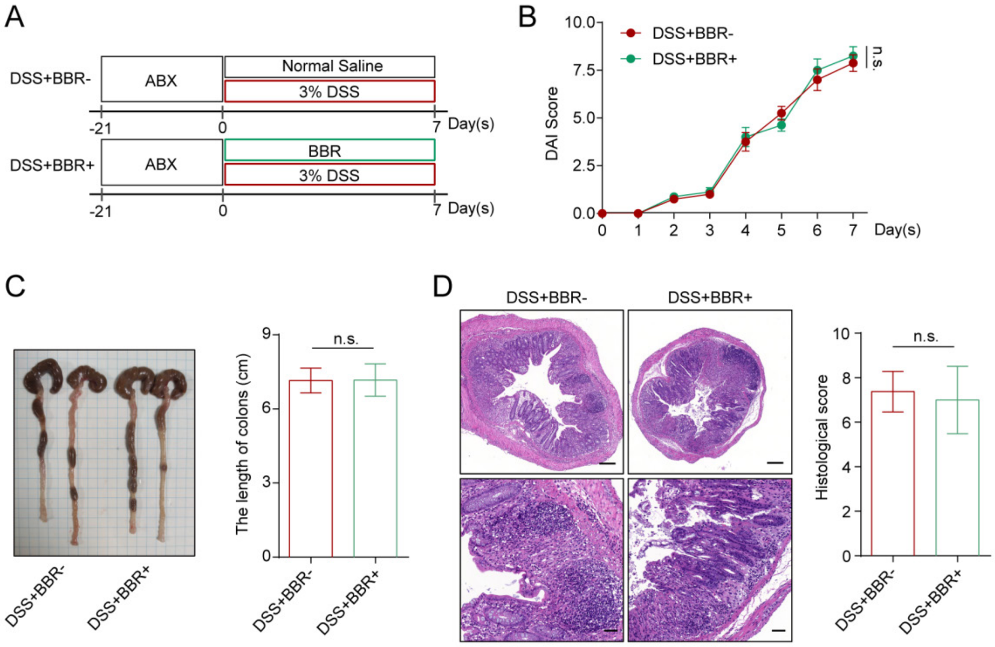
A
DSS+BBR-
ABX
Normal Saline
3% DSS
-21
0
7
Day(s)
DSS+BBR+
ABX
BBR
3% DSS
-21
0
7
Day(s)
B
DSS+BBR-
DSS+BBR+
DAI Score
n.s.
Day(s)
C
DSS+BBR-
DSS+BBR+
The length of colons (cm)
n.s.
D
DSS+BBR-
DSS+BBR+
Histological score
n.s.

Figure S3

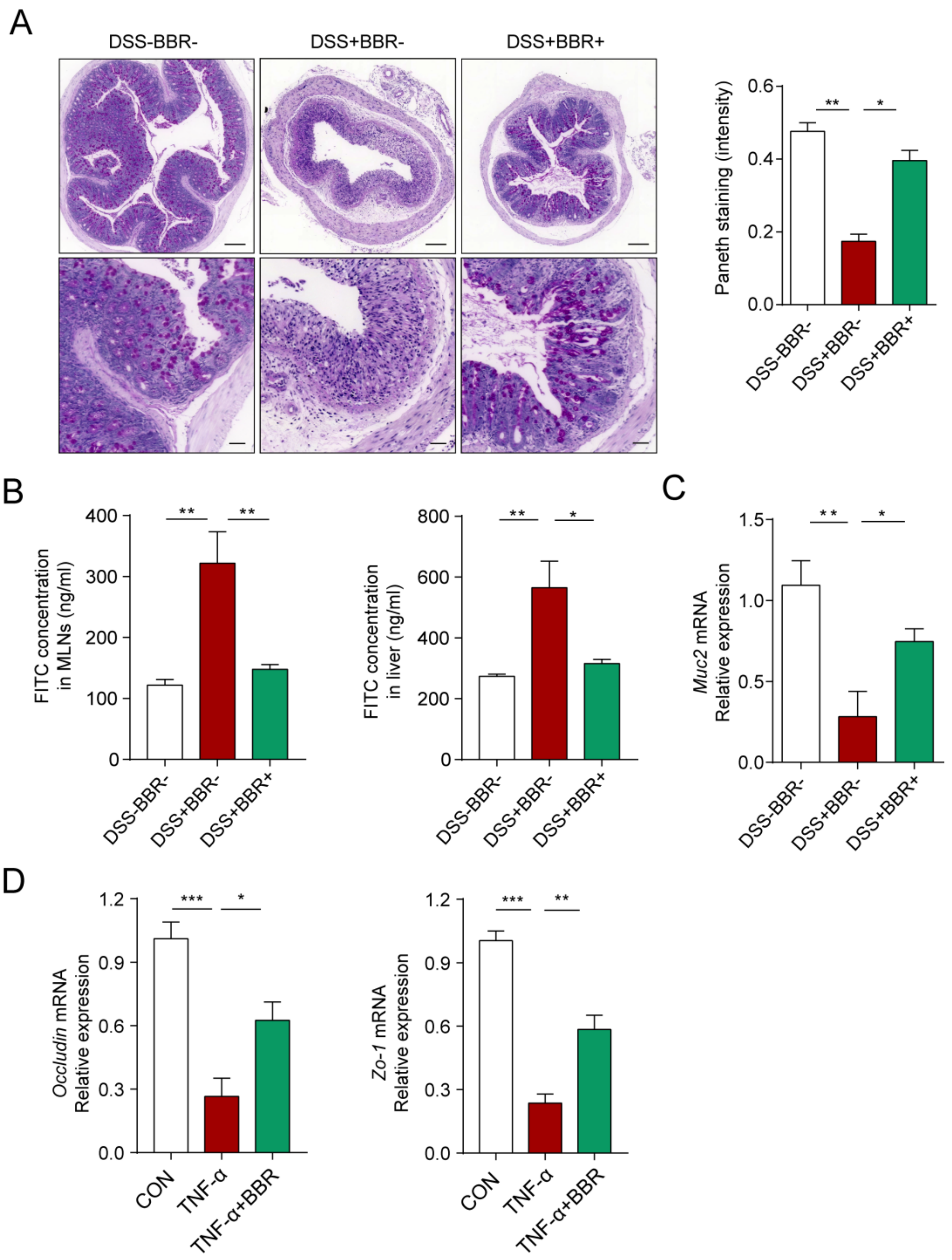
A
DSS-BBR-
DSS+BBR-
DSS+BBR+
Paneth staining (intensity)
**
*
B
FITC concentration in MLNs (ng/ml)
FITC concentration in liver (ng/ml)
C
Muc2 mRNA Relative expression
D
Occludin mRNA Relative expression
Zo-1 mRNA Relative expression
CON
TNF-α
TNF-α+BBR
***

Figure S4

A

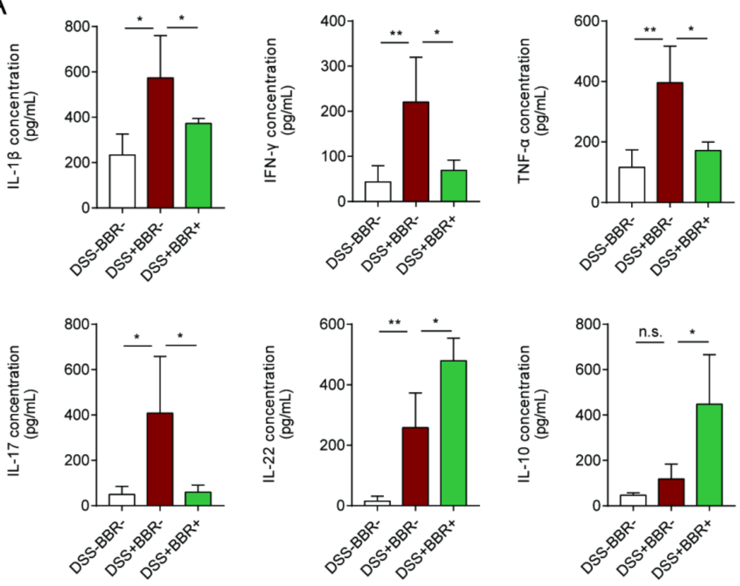

IL-1β concentration (pg/mL)
IFN-γ concentration (pg/mL)
TNF-α concentration (pg/mL)
IL-17 concentration (pg/mL)
IL-22 concentration (pg/mL)
IL-10 concentration (pg/mL)
DSS-BBR-
DSS+BBR-
DSS+BBR+
n.s.

Figure S5

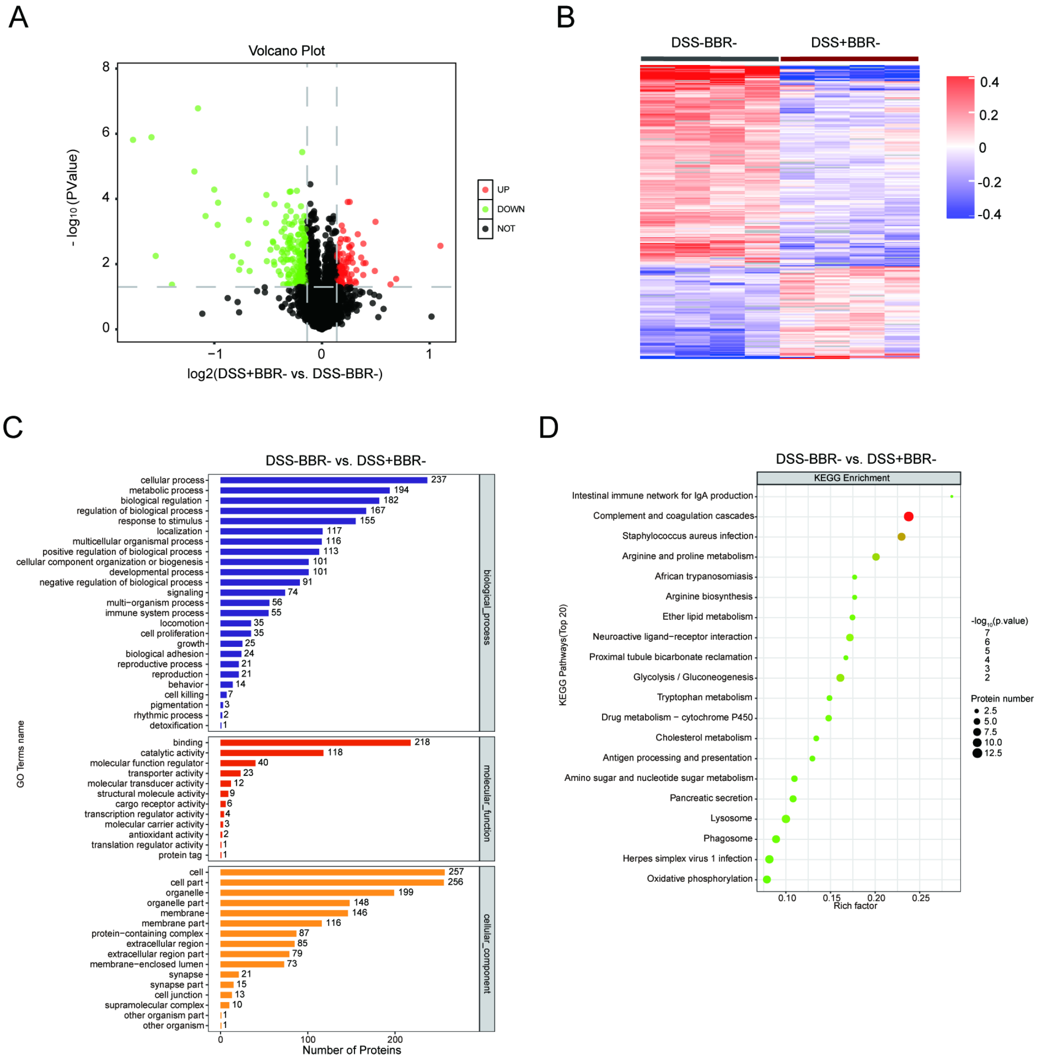

A
Volcano Plot
- log10 (PValue)
log2(DSS+BBR- vs. DSS-BBR-)
UP
DOWN
NOT
B
DSS-BBR-
DSS+BBR-
C
DSS-BBR- vs. DSS+BBR-
GO Terms name
Number of Proteins
biological_process
molecular_function
cellular_component
cellular process 237
metabolic process 194
biological regulation 182
regulation of biological process 167
response to stimulus 155
localization 117
multicellular organismal process 116
positive regulation of biological process 113
cellular component organization or biogenesis 101
developmental process 101
negative regulation of biological process 91
signaling 74
multi−organism process 56
immune system process 55
locomotion 35
cell proliferation 35
growth 25
biological adhesion 24
reproductive process 21
reproduction 21
behavior 14
cell killing 7
pigmentation 3
rhythmic process 2
detoxification 1
binding 218
catalytic activity 118
molecular function regulator 40
transporter activity 23
molecular transducer activity 12
structural molecule activity 9
cargo receptor activity 6
transcription regulator activity 4
molecular carrier activity 3
antioxidant activity 2
translation regulator activity 1
protein tag 1
cell 257
cell part 256
organelle 199
organelle part 148
membrane 146
membrane part 116
protein−containing complex 87
extracellular region 85
extracellular region part 79
membrane−enclosed lumen 73
synapse 21
synapse part 15
cell junction 13
supramolecular complex 10
other organism part 1
other organism 1
D
DSS-BBR- vs. DSS+BBR-
KEGG Enrichment
KEGG Pathways(Top 20)
Rich factor
Intestinal immune network for IgA production
Complement and coagulation cascades
Staphylococcus aureus infection
Arginine and proline metabolism
African trypanosomiasis
Arginine biosynthesis
Ether lipid metabolism
Neuroactive ligand−receptor interaction
Proximal tubule bicarbonate reclamation
Glycolysis / Gluconeogenesis
Tryptophan metabolism
Drug metabolism − cytochrome P450
Cholesterol metabolism
Antigen processing and presentation
Amino sugar and nucleotide sugar metabolism
Pancreatic secretion
Lysosome
Phagosome
Herpes simplex virus 1 infection
Oxidative phosphorylation
-log10(p.value)
Protein number
